# Humic Substances Mediate Anaerobic Methane Oxidation Linked to Nitrous Oxide Reduction in Wetland Sediments

**DOI:** 10.3389/fmicb.2020.00587

**Published:** 2020-04-15

**Authors:** Edgardo I. Valenzuela, Claudia Padilla-Loma, Nicolás Gómez-Hernández, Nguyen E. López-Lozano, Sergio Casas-Flores, Francisco J. Cervantes

**Affiliations:** ^1^División de Ciencias Ambientales, Instituto Potosino de Investigación Científica y Tecnológica, San Luis Potosí, Mexico; ^2^División de Biología Molecular, Instituto Potosino de Investigación Científica y Tecnológica, San Luis Potosí, Mexico; ^3^Laboratory for Research on Advanced Processes for Water Treatment, Engineering Institute, Campus Juriquilla, Universidad Nacional Autónoma de México, Querétaro, Mexico

**Keywords:** greenhouse gases, anaerobic methanotrophy, wetlands, nitrous oxide, extracellular electron transfer, natural organic matter, archaea, denitrification

## Abstract

Humic substances are redox-active organic molecules, which play pivotal roles in several biogeochemical cycles due to their electron-transferring capacity involving multiple abiotic and microbial transformations. Based on the redox properties of humic substances, and the metabolic capabilities of microorganisms to reduce and oxidize them, we hypothesized that they could mediate the anaerobic oxidation of methane (AOM) coupled to the reduction of nitrous oxide (N_2_O) in wetland sediments. This study provides several lines of evidence indicating the coupling between AOM and the reduction of N_2_O through an extracellular electron transfer mechanism mediated by the redox active functional groups in humic substances (e.g., quinones). We found that the microbiota of a sediment collected from the Sisal wetland (Yucatán Peninsula, southeastern Mexico) was able to reduce N_2_O (4.6 ± 0.5 μmol N_2_O g _sed._^–1^ day^–1^) when reduced humic substances were provided as electron donor in a close stoichiometric relationship. Furthermore, a microbial enrichment derived from the wetland sediment achieved simultaneous ^13^CH_4_ oxidation (1.3 ± 0.1 μmol ^13^CO_2_ g _sed._^–1^ day^–1^) and N_2_O reduction (25.2 ± 0.5 μmol N_2_O g _sed._^–1^ day^–1^), which was significantly dependent on the presence of humic substances as an extracellular electron shuttle. Taxonomic characterization based on 16S rRNA gene sequencing revealed *Acinetobacter* (a ɣ-proteobacterium), the Rice Cluster I from the *Methanocellaceae* and an uncultured archaeon from the *Methanomicrobiaceae* family as the microbes potentially involved in AOM linked to N_2_O reduction mediated by humic substances. The findings reported here suggest that humic substances might play an important role to prevent the emission of greenhouse gases (CH_4_ and N_2_O) from wetland sediments. Further efforts to evaluate the feasibility of this novel mechanism under the natural conditions prevailing in ecosystems must be considered in future studies.

## Introduction

Continuous emissions of greenhouse gases (GHG), such as carbon dioxide (CO_2_), methane (CH_4_), and nitrous oxide (N_2_O) have been associated to several environmental problems that include global warming (GW), alterations of precipitation patterns, changes in groundwater levels and soil conditions, as well as extreme weather events (Kumar et al., 2020). Therefore, intensive research is currently underway to elucidate the microbial and abiotic processes driving these GHG emissions from natural environments.

Wetlands are highly dynamic ecosystems that, collectively, constitute the largest biogenic source of GHG (Turetsky et al., 2014). For instance, the net amount of CH_4_ released from these environments represents one third of the global CH_4_ budget (∼164 Tg yr^–1^) (Bridgham et al., 2013). Regarding N_2_O, the global emissions estimation from coastal wetlands is up to 4.8 Tg N year^–1^ and this amount could be further increased due to anthropogenic exacerbation of the N cycle (Murray et al., 2015). Altogether, CH_4_ and N_2_O are two of the most hazardous GHG, both because of their high GW potential (25 and 300 times higher than that of CO_2_, respectively), and because of their long residence time in the Earth’s atmosphere (12 and 114 years, respectively) (Tangen and Bansal, 2019).

Anaerobic degradation of natural organic matter (NOM) by microorganisms, which involves the methanogenesis process, constitutes an important source for CO_2_ and CH_4_ emissions from aquatic ecosystems (Gougoulias et al., 2014; Laruelle et al., 2014; Lever, 2016). In the same fashion, N_2_O is produced by anaerobic microbes in nitrogen-rich environments due to incomplete denitrification, and through the nitrification process occurring in the oxic-anoxic interfaces present above and below oxygen deficient zones (Babbin et al., 2015; Ji et al., 2015; Pajares and Ramos, 2019).

As a counterpart of these microbial sources of GHG emissions, there are several mechanisms for CH_4_ and N_2_O microbial uptake, which have been extensively described. Regarding CH_4_, after assuming for decades that only aerobic microbes could oxidize this very stable compound (via a monooxygenase activation), several inorganic terminal electron acceptors (TEAs) have been reported to support anaerobic methane oxidation (AOM) by specialized anaerobic microorganisms (Gupta et al., 2013; Segarra et al., 2015). These TEAs include sulfate (SO_4_^2–^), nitrate and nitrite (NO_3_^–^, NO_2_^–^), as well as metallic oxides of iron [Fe(III)] and manganese [Mn(IV)] (Welte et al., 2016; He et al., 2018; Bhattarai et al., 2019). Recently, the redox-active fraction of the continuously decaying NOM, commonly referred to as humic substances (Lehmann and Kleber, 2015), as well as their structural analogs (e.g., quinones), have also been found to be suitable TEAs for achieving AOM (Scheller et al., 2016; Valenzuela et al., 2017; Bai et al., 2019). Moreover, humic substances can promote AOM not only by acting as TEA, but also by shuttling electrons derived from AOM toward metallic oxides reduction (Smemo and Yavitt, 2011; He et al., 2019; Valenzuela et al., 2019).

Concerning N_2_O, the only known microbial process responsible for its consumption involves its reduction to molecular nitrogen (N_2_). This one-step transformation is achieved by *nosZ* gene (nitrous oxide reductase) bearing microorganisms, which might not be mandatorily denitrifiers (Hallin et al., 2018). Regularly, the source of electrons for this reaction comes from labile molecules in NOM. Additionally, it has recently been reported that CH_4_ could serve as an electron donor for this reaction, implying the existence of microorganisms capable of coupling the simultaneous consumption of two GHG (Cheng et al., 2019). Despite this, the underlying mechanisms and the main environmental drivers of this process remain unknown. Taking into account the previous evidence showing that reduced humic substances could serve as electron donor for denitrification (Aranda-Tamaura et al., 2007; van Trump et al., 2011), and that oxidized humic substances could support AOM by serving as TEA (Valenzuela et al., 2017), we aimed to decipher if they could mediate AOM linked to N_2_O reduction via an inter-species electron transfer (IET) process. It has previously been proven that humic substances and other quinone-containing materials (such as biochar or activated carbon) may link the oxidation and reduction of molecules that one single microorganism could not accomplish due to metabolic limitations (Liu et al., 2012; Lovley, 2017; Rotaru et al., 2018).

## Materials and Methods

### Wetland and Sediment Sampling Description

The Sisal wetland is located in the coastal zone of the Yucatán Peninsula (southeastern Mexico, 21°09′26′′N, 90°03′09′′W). This marsh possesses a semi-arid climate, a high degree of karstification, as well as intermittent saltwater inputs from the ocean causing variable salinity levels (Batllori-Sampedro et al., 1999). Sediment cores were collected from the wetland in January 2016. The cores were sampled under a water column of approximately 70 cm in depth and the length of the cores was 15 cm. All sediment and water samples were stored in tight sealed plastic containers, which were maintained in ice until their arrival to the laboratory where they were then stored at 4°C in a dark room for 18 months before conducting the incubations. Before performing the incubation assays, sediment and its pore water were chemically characterized. Some of the most relevant chemical components found in the collected water column samples and extracted pore water were sulfate and nitrate due to its potential role as electron acceptors for microbial activity. Potential electron donors identified were sulfide and hints of degradable NOM detected as total organic carbon (TOC). Further details on these characteristics have previously been reported elsewhere (Valenzuela et al., 2017, 2019).

### Microcosms Set-Up

#### Kinetics of N_2_O Reduction

An initial evaluation of the capacity of the wetland sediment biota to employ reduced Pahokee Peat humic substances (PPHS, catalog number from the IHSS: 1S103H) as electron donors for N_2_O reduction was performed. To this end, serum bottles (25 mL) were inoculated with 1 g of previously homogenized wetland sediment, and 15 mL of inorganic basal medium enriched with PPHS at a concentration of 1 g L^–1^ was employed. The composition of the basal medium used (in g L^–1^) was as follows (modified from Cervantes et al., 2000): NaHCO_3_ (5), NH_4_Cl (0.3), K_2_HPO_4_ (0.2), MgCl_2_ 6H_2_O (0.03), and CaCl_2_ (0.1). Trace elements were included in the medium by adding 1 mL L^–1^ of a solution with the following composition (in mg L^–1^): FeCl_2_⋅4H_2_O (2,000), H_2_BO_3_ (50), ZnCl_2_ (50), CuCl_2_⋅6H_2_O (90), MnCl_2_⋅4H_2_O (500), AlCl_3_⋅6H_2_O (90), CoCl_2_⋅6H_2_O (2,000), NiCl⋅6H_2_O (920), Na_2_SeO⋅5H_2_O (162), (NH_4_)_6_Mo_7_O_24_ (500), EDTA (1,000), Na_2_WO_4_⋅H_2_O (100), and 1 mL L^–1^ HCl at 36%. NaCl (3 g L^–1^) was added to the medium to match the salinity level detected in the water column at the moment of the sediment sampling (Valenzuela et al., 2017). The final pH of the medium was 7.2 ± 0.05 and it remained constant throughout the incubation period. Controls lacking PPHS were also included, as well as PPHS enriched sterilized controls, which were prepared by autoclaving (three cycles) and subsequent addition of anhydrous chloroform (99%, Sigma-Aldrich) at a concentration of 10% v/v. All microcosms were incubated under anoxic conditions for 2 months with hydrogen (H_2_) as the electron donor to achieve PPHS reduction (these treatments are referred to as PPHS_red_). The headspace of all microcosms was flushed with argon (99.9% purity, Praxair) for 10 min, and then H_2_ was provided to a partial pressure of 0.67 atm with disposable syringes (supply of H_2_ was done three times during the reduction process). After this incubation period to achieve PPHS reduction, all bottles were thoroughly flushed with Ar for 1 h to remove the remaining H_2_, which was confirmed by chromatographic measurements, and then the electrons stored as PPHS_red_ were measured by the ferrozine technique (Lovley et al., 1996; Valenzuela et al., 2017).

Controls containing the same concentration of oxidized PPHS (PPHS_ox_) were prepared as follows: bottles pre-incubated as previously described, but containing only sediment, were provided with PPHS_ox_ from a concentrated stock prepared by magnetic stirring using the same inorganic medium previously described. Prior to spiking the microcosms with the PPHS_ox_, dissolved oxygen was flushed away from the stock solution by purging with Ar for 1 h. The purpose of preparing these controls in this manner was to attenuate the sediment intrinsic electron acceptors and donors in the same way, and during the same period, as in the main treatments (those including PPHS and provided with H_2_ for their reduction). To begin the N_2_O reduction experiment, 4 mL of N_2_O were spiked to all microcosms, except to those PPHS_red_ bottles serving as endogenous controls (to verify the re-oxidation of PPHS_red_ by intrinsic TEAs remaining). Afterward, the zero-time gaseous measurements were done, and the incubation period was started by placing all bottles in a dark room at 28°C, which was the temperature prevailing in the wetland at the moment of sampling. The incubation was carried out without mechanical shaking. The number of replicates per treatment was three (detailed description concerning this experimental setting is provided in Supplementary Table S1).

A preliminary incubation was conducted with PPHS_red_ and ^15^N_2_O to verify the reduction of ^15^N_2_O to ^15^N_2_, which was confirmed by GC-MS analysis (see Supplementary Figure S1). Therefore, N_2_O consumption was referred to as N_2_O reduction in the present study.

#### Kinetics of Simultaneous N_2_O and ^13^CH_4_ Consumption

One gram of homogenized wetland sediment was inoculated into 25 mL serum bottles containing 15 mL of the anoxic basal medium previously described. Afterward, the headspace of each bottle was flushed with argon gas (Ar) for 10 min. All microcosms were then pre-incubated in a dark room at 28°C for approximately 30 days. The purpose of this initial incubation was to deplete endogenous electron donors and acceptors, such as labile organic molecules, sulfate, nitrate and oxidized metals, which were in fact already subject to attenuation due to the potential microbial activity taking place during the storage period even under the refrigeration temperature (18 months of storage at 4°C from sampling to incubation). After this incubation period, microcosms were taken inside an anoxic chamber (COY 14500; atmosphere composed of N_2_/H_2_, 95%/5% v/v) to replace the liquid phase by freshly prepared anoxic basal medium enriched with 500 mg L^–1^ of PPHS. Control incubations were filled with regular basal medium lacking PPHS. Once the basal medium was replaced, all microcosms were sealed with rubber stoppers and aluminum crimps, taken outside the anaerobic chamber and their atmosphere was flushed with Ar for 10 min. Once these anoxic microcosms were prepared, 2 mL of ^13^CH_4_ (99 atom.%, Sigma-Aldrich) and/or 4 mL of N_2_O (99.9% purity, Sigma-Aldrich) were injected into the bottles’ headspace using plastic disposable syringes. Endogenous controls were left without addition of GHG, while sterile controls were prepared as described above. The number of replicates per treatment was three (detailed description concerning this experimental design is provided in Supplementary Table S2).

The first incubation cycle in which microbial ^13^CH_4_ oxidation and N_2_O reduction was observed lasted 9 days. After this period, microcosms were supplied with new basal medium (including fresh PPHS where appropriate) inside the anaerobic chamber and then flushed with Ar for 10 min. ^13^CH_4_ and N_2_O were spiked again, and a second incubation cycle of 9 days was started.

### Analytical Techniques

#### Sulfate, Sulfide, Nitrate, and Nitrite Measurements

The concentrations of SO_4_^2–^, dissolved sulfide (HS^–^), NO_3_^–^ and NO_2_^–^ were measured according to standard methodologies previously established (capillary electrophoresis and spectrophotometric detection) (Cord-Ruwisch, 1985; Soga and Ross, 1999; APHA/AWWA/WEF, 2012). A detailed description of these methodologies and their modifications can be found in Rios-Del Toro et al. (2018a).

### Isotopic Carbon Dioxide and Nitrous Oxide Determinations

Simultaneous quantification of ^13^CO_2_ production from ^13^CH_4_ oxidation and N_2_O consumption was conducted by mass spectrometry (MS) (Agilent Technologies 5977A Series MSD) complemented by 7890B gas chromatograph (GC). Separation was achieved with a HP-PLOT/Q + PT capillary column (30 m × 0.320 mm ID × 0.20 μm) from Agilent Technologies. Helium was used as carrier gas at 0.3 mL min^–1^. The chromatographic method was as follows: the starting temperature was 70°C, and then a ramp with an increase of 20°C per min was implemented for 3 min. The temperatures of injector and MS source were maintained at 250 and 230°C, respectively. The injection volume was 20 μL and there was only one replicate of injection per analyzed sample. The gas injected into the gas chromatograph was manually taken directly from the headspace of the incubations and immediately injected into the GC port. This chromatographic method was also adequate for the detection of ^12^CH_4_, which was also monitored throughout the experiments.

### Quantification of Electron-Donating Capacity in Slurry Samples

The reduction of humic material in the form of PPHS or intrinsic NOM was assessed as the amount of ferrous iron produced by the reaction of ferric citrate with slurry taken from the microcosms under anoxic conditions. The ferrous iron released was then measured by the ferrozine technique (Lovley et al., 1996) and corrected for intrinsic ferrous iron detected (Fe^2+^ measured in samples after acid treatment without addition of ferric citrate) (Stookey, 1970). These measurements were performed in a spectrophotometer located inside the COY 14500 anaerobic chamber previously described. Further details on this methodology have been previously described (Valenzuela et al., 2017).

### Molecular Analysis

#### DNA Extraction

Two replicates for each experimental treatment were sacrificed at the end of the incubation periods for total DNA extraction. Bottles were vigorously shaken and then 500 μL of slurry were taken with sterile disposable syringes to extract DNA using the PowerSoil DNA extraction kit (Mo Bio Laboratories, Carlsbad, CA, United States) according to the protocol described by the manufacturer. The construction of the 16S rRNA genomic libraries was based on DNA samples processed in an independent manner in order to obtain parallel sequencing results for each experimental replicate.

### Sequencing and Genomic Libraries Construction

Total DNA isolated from each experimental replicate was amplified using primers targeting the 16S rRNA gene of Bacteria (V3–V4 region, 341F-805R) and Archaea (340F-1000R) (Gantner et al., 2011), both fused with Illumina adapter overhang nucleotide sequences. PCRs for bacterial 16S rRNA region were performed in 25 μL reaction mixtures using Invitrogen HF Platinum Taq Polymerase (Thermo Fisher Scientific, United States) under the following conditions: denaturation at 95°C for 90 s, followed by 30 cycles of amplification at 95°C for 15 s, 57°C for 30 s, 72°C for 30 s, 80°C for 30 s and finished with 95°C for 15 s and 60°C for 10 s. PCRs for archaeal 16S rRNA region were performed under the conditions reported by Gantner et al. (2011). PCR products were indexed using Nextera XT Index Kit v2 (Illumina, San Diego, CA, United States) according to the Illumina’s 16S Metagenomic Sequencing Library Preparation protocol. Libraries were further sequenced by single end with Illumina MiSeq sequencer.

### 16S rRNA Bioinformatic Analysis

Mothur open source software (v 1.34.4) was used for analysis of 16S rRNA libraries (Kozich et al., 2013). Sequences with a length less than 500 bp, homopolymer runs of eight or more bases, those with more than one mismatch to the sequencing primer and Q-value average below 25 were discarded. The potential occurrence of chimeric sequences was analyzed using UCHIME algorithm. Group membership was determined prior to trimming of the barcode and primer sequence. A distance matrix was calculated across the set of non-redundant sequences and the readings were grouped into operational taxonomic units (OTUs) with a similarity threshold of 97%. Mothur’s Bayesian classifier and the SILVA v.132 reference set were used to taxonomically categorize the sequences using the nearest alignment space termination (NAST) algorithm. Taxonomic assignments were made with a confidence threshold greater than 80% of bootstrap value. The accession numbers of sequences in this work were deposited in the GenBank sequence read archive under the BioProject with PRJNA576687 accession number.

## Results

### Kinetics of N_2_O Reduction With PPHS_red_ as Electron Donor

**FIGURE 1:**
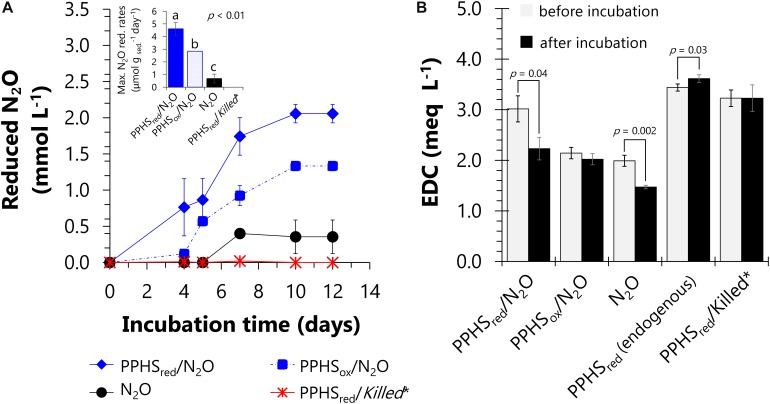
Nitrous oxide reduction linked to re-oxidation of reduced functional groups in reduced Pahokee Peat Humic Substances (PPHS_red_). Panel **(A)** depicts the normalized (initial concentration – concentration, C_i_–C) kinetics of N_2_O reduction. The inset shows the maximum rates of N_2_O reduction based on the linear regressions of at least three sampling points during the period of highest activity. Statistically different groups (rates) are represented with letters obtained via a one-way ANOVA and the Duncan *post hoc* test (95% percent confidence interval). Panel **(B)** shows changes on the electron donating capacity (EDC) of PPHS or intrinsic NOM before and after incubation with or without N_2_O after 12 days of incubation. Significant changes in EDC during incubation time are denoted with *p*-values (< 0.05), which were evaluated through a Student’s *t*-test (95% percent confidence interval, degrees of freedom = 4). Data represent the average from triplicate incubations ± standard error. *Killed controls contained the same concentration of N_2_O as in the main experimental treatments (3.23 ± 0.03 mmol L^– 1^). Detailed description of the experimental set-up employed in this experiment is shown in Supplementary Table S1.

^∗^Further details on these thermodynamic calculations are included in Supporting Information (Supplementary Figure S3).

The microbial communities present in the sediments of Sisal wetland have previously been shown to perform AOM linked to PPHS reduction (Valenzuela et al., 2017). However, their capacity to use PPHS_red_ as electron donor for N_2_O reduction has not been demonstrated. With the purpose of verifying this unexplored process, sediment incubations were conducted including PPHS_red_ and N_2_O, along with the respective controls (see Supplementary Table S1). In an initial incubation period lasting 12 days, the wetland sediment’s microbiota achieved maximum N_2_O reducing activities of up to 4.6 ± 0.5 μmol g _sed_.^–1^ day^–1^ [mean ± standard error (SE), *n* = 3] in microcosms enriched with PPHS_red_ (Figure 1A). The oxidation of 0.8 ± 0.3 milli-equivalents (meq) L^–1^ (mean ± SE, *n* = 3) derived from PPHS_re__d_ occurred in parallel to N_2_O reduction in this treatment during the whole incubation period (Figure 1A). This PPHS_red_ oxidation activity was calculated by the loss on their EDC through the same incubation period (Figure 1B). Considering quinones/hydroquinones as the main redox groups in humic substances (Scott et al., 1998), the stoichiometry of N_2_O reduction coupled to hydroquinones oxidation can be considered as follows:

(1) $${\text{N}}_{2}\text{O + }{\text{QH}}_{2}\;\cdot \;{\text{PPHS}}_{\text{red}}\;\to \;{\text{N}}_{2}\;+\text{ Q }\cdot \;{\text{PPHS}}_{\text{ox}}\;+\;{\text{H}}_{2}\text{O }\Delta {\text{G}}^{\text{ο}’}\;(\text{kJ }{\text{mol}}^{-1})\;=\;-814\text{ to }-1276*$$

Where QH_2_-PPHS_red_ refers to reducing equivalents stored as hydroquinones in PPHS_red_ (2 reducing equivalents per hydroquinone moiety) and Q-PPHS_ox_ represents quinone equivalents produced as PPHS_ox_ during the oxidation of QH_2_-PPHS_red_. The ratio of QH_2_-PPHS_red_ oxidized: N_2_O reduced obtained at the end of the experiments was approximately 1.08 (corrected for the oxidation of PPHS_red_ quantified in controls lacking N_2_O and for the N_2_O reduction measured in controls amended with PPHS_ox_, respectively) (Figure 1 and Table 1), which is in agreement with the expected stoichiometric value (1:1, Eq. 1).

**TABLE 1:** Electron balance during the reduction of N_2_O with reduced Pahokee Peat Humic Substances (PPHS_red_) as electron donors.

Treatment	N_2_O reduced [mmol L^–1^]^e^	QH_2_-equivalents^f^ oxidized (intrinsic NOM or PPHS) [mmol L^–1^]^g^	Molar ratio N_2_O reduced:QH_2_ oxidized^i^
N_2_O (only sediment)^a^	0.4 ± 0.2	0.52 ± 0.07	1:0.67
PPHS_red_ (endogenous)^b^	n.a.	n.d.^h^	n.a.
PPHS_red_/N_2_O	2.06 ± 0.01	0.8 ± 0.3	1:1.08
PPHS_ox_/N_2_O^c^	1.33 ± 0.01	n.d.^h^	n.a.
PPHS_red_/N_2_O-*Killed*^d^	n.d.	n.d.^h^	n.a.

*n.d., not detected; n.a., not applicable. ^*a*^Only sediment refers to those controls spiked with N_2_O but lacking added PPHS. ^*b*^Endogenous refers to experimental controls lacking added N_2_O as terminal electron acceptor but including PPHS_*red*_. ^*c*^Experimental treatment including oxidized PPHS (not reduced prior to the incubation with N_2_O). ^*d*^Killed controls’ headspace was spiked with the same volumes of N_2_O added in the microbially active treatments. ^*e*^N_2_O consumed after the whole incubation period. ^*f*^Hydroquinone equivalents (see Eq. 1). ^*g*^Loss of electron donating capacity (EDC) due to hydroquinones re-oxidation linked to N_2_O reduction. Values corrected for Fe^2+^ intrinsic to the wetland sediment detected by the Ferrozine technique. ^*h*^Loss of EDC during incubation not statistically significant (see Figure 1B). ^*i*^Molar ratio calculated by correcting the value of N_2_O depleted in the PPHS_*red*_ treatment with that of the N_2_O consumption detected in the PPHS_*ox*_ treatment.*

Furthermore, the oxidation of reduced redox groups in the intrinsic NOM present in the wetland sediment (incubated without PPHS), was concomitant to the N_2_O reduction observed in these controls (the ratio QH_2_-NOM_red_ oxidized:N_2_O reduced was 0.67, corrected for the corresponding controls). This confirms the active role of reduced groups in the intrinsic NOM of the wetland sediment to sustain the N_2_O reducing process (Figure 1 and Table 1). This is in agreement with previous studies showing that the electron accepting capacity (EAC) of the intrinsic NOM in the same sediment partially sustained AOM by serving as TEA (Valenzuela et al., 2017). Namely, redox-active moieties present in intrinsic NOM play an active role in microbial activities related to the consumption of GHG. Additionally, substantial N_2_O reduction occurred in sediment incubations spiked with PPHS_ox_ (2.8 μmol g_sed._^–1^ day^–1^), which was significantly higher than that measured in the absence of PPHS (0.7 ± 0.3 μmol g_sed._^–1^ day^–1^) (mean ± SE, *n* = 3). This suggests that the labile fraction of PPHS_ox_ partly fueled N_2_O reduction. Sediment incubations enriched with PPHS_red_, but lacking added N_2_O (endogenous controls) did not show any significant loss on the initial EDC (including both PPHS_red_ and reduced intrinsic NOM), suggesting that N_2_O was the only TEA fueling microbial PPHS_red_ oxidation (Figure 1B). Sulfate, nitrite and nitrate were not detected during these incubations further confirming that N_2_O was the most relevant TEA. Abiotic controls (killed microorganisms) including both PPHS_red_ and N_2_O did not show any activity (Table 1), validating the biological nature of these redox processes.

### AOM Linked to N_2_O Reduction Mediated by Humic Substances

#### N_2_O Reduction Rates

**FIGURE 2:**
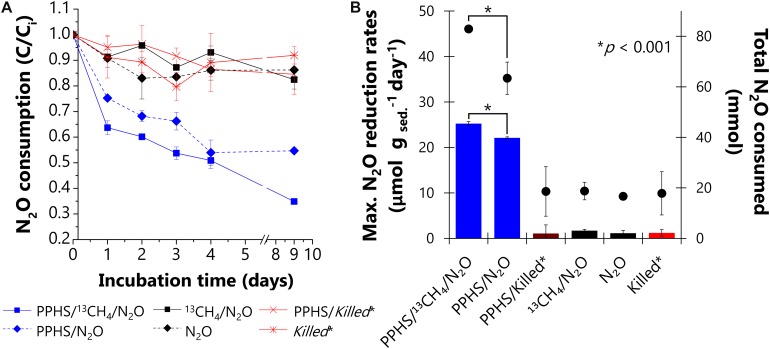
Nitrous oxide reduction promoted by Pahokee Peat Humic Substances (PPHS) acting as electron shuttle and ^13^CH_4_ as electron donor during the second cycle of incubation. Panel **(A)** depicts the normalized (concentration/initial concentration, C/C_i_) kinetics of N_2_O consumption with and without PPHS as electron shuttle. Panel **(B)** shows the maximum N_2_O reduction rates (bars, left axis) based on the linear regressions of at least three sampling points during the period of the highest activity. The net amount of N_2_O depleted after 9 days of incubation is shown in the right axis (**⋅** symbols). Data represent the average from triplicate incubations ± standard error. *Killed controls contain the same concentration of ^13^CH_4_ (∼4 mmol L^– 1^) and N_2_O (6.6 ± 0.4 mmol L^– 1^) as in the main experimental treatments. Significant differences in the maximum N_2_O consumption rates and total N_2_O reduced during incubation time among the treatments containing PPHS are indicated with asterisks denoting *p*-values (<0.001), which were evaluated through a Student’s *t*-test (95% percent confidence interval, degrees of freedom = 4).

After a conditioning pre-incubation cycle, intended to exhaust intrinsic electron donors and acceptors present in the wetland sediment, N_2_O reduction was documented in two subsequent incubation periods (referred to as 1st and 2nd incubation cycles). During the 1st cycle, high N_2_O reduction rates were observed only in microcosms enriched with PPHS (0.23 ± 0.03 and 0.15 ± 0.03 mmol N_2_O g_sed._^–1^ day^–1^ (mean ± SE, *n* = 3) in the presence and in the absence of ^13^CH_4_, respectively). These treatments displayed nearly complete depletion of the supplied N_2_O within the first 3 days of incubation (Supplementary Figure S2). However, this activity was mainly fueled by the oxidation of labile organic compounds originally present in the wetland sediment. Thus, the role of PPHS as redox mediator linking AOM to N_2_O reduction was not conclusive in the 1st cycle (Supplementary Figure S2). Nevertheless, throughout the 2nd incubation cycle (Figure 2), the importance of PPHS fueling the consumption of both GHG was explicitly shown by ∼30% more N_2_O reduced in the treatment containing ^13^CH_4_ as electron donor. The maximum reduction rate observed in this treatment was 25.5 ± 0.5 μmol N_2_O g _sed._^–1^ day^–1^ (mean ± SE, *n* = 3), 15% higher than the rate observed in experimental controls containing PPHS, but lacking ^13^CH_4_ (22.1 ± 0.2 μmol N_2_O g _sed._^–1^ day^–1^) (mean ± SE, *n* = 3). Marginal rates of N_2_O reduction (1.7 ± 0.3 and 1.2 ± 0.6 μmol N_2_O g _sed._^–1^ day^–1^) (mean ± SE, *n* = 3) were observed with and without ^13^CH_4_ addition, respectively, in microcosms lacking PPHS, which were in fact very similar to the activity observed in abiotic controls (Figure 2B). Moreover, high N_2_O reduction was only observed in incubations amended with PPHS or with PPHS/^13^CH_4_ (Figure 2A).

### AOM and Its Coupling With N_2_O Reduction

**FIGURE 3:**
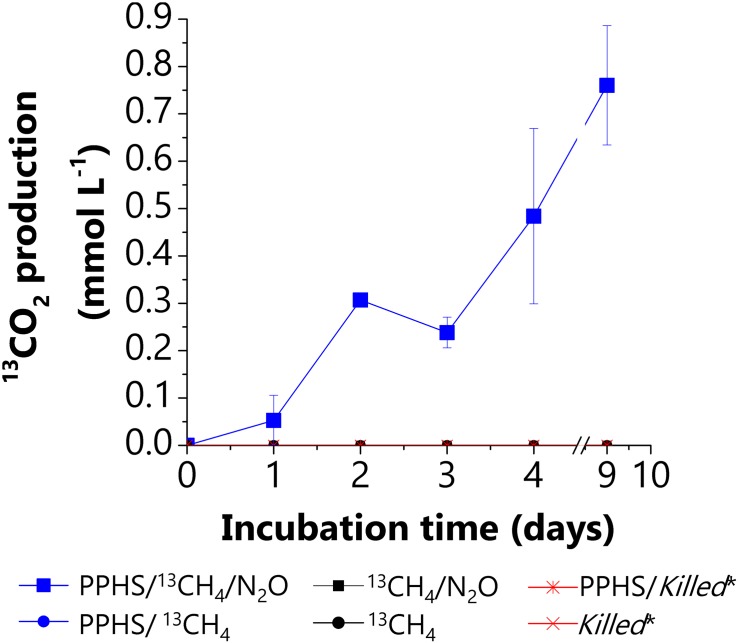
Anaerobic ^13^CH_4_ oxidation measured as ^13^CO_2_ production from AOM linked to N_2_O reduction via electron shuttling mediated by PPHS. Data represent the average from triplicate incubations ± standard error.

^∗^Further details on these thermodynamic calculations are included in Supporting Information (Supplementary Figure S3).

Supplementary evidence demonstrating the coupling between AOM and N_2_O reduction was obtained by quantifying the amount of ^13^CO_2_ derived from ^13^CH_4_ oxidation in all experimental conditions (Figure 3). During the 2nd incubation cycle, ^13^CO_2_ production was only detected in sediment incubations performed with ^13^CH_4_/N_2_O/PPHS (1.3 ± 0.1 μmol ^13^CO_2_ g _sed._^–1^ day^–1^, Figure 3) (mean ± SE, *n* = 3). Sulfide prevailed at very low concentrations during these incubations (<5 μM), thus its contribution to N_2_O reduction was negligible. Nevertheless, the labile fraction present in PPHS fueled significant N_2_O reduction in the treatment N_2_O/PPHS, which obstructed the accurate assessment of the stoichiometry of the process. At the end of the incubation period, the amount of N_2_O reduced in the treatment ^13^CH_4_/N_2_O/PPHS was 2.6 ± 0.4 meq L^–1^ (mean ± SE, *n* = 3, corrected for the N_2_O reduction observed in the control treatment, N_2_O/PPHS) and the produced ^13^CO_2_ was equivalent to 6.1 ± 0.6 meq L^–1^ (mean ± SE, *n* = 3). Thus, the ratio N_2_O reduced to ^13^CO_2_ produced was 0.43, which means that N_2_O reduction accounted for 43% of the ^13^CH_4_ oxidized (quantified as ^13^CO_2_). These calculations were based on the following stoichiometry:

(2) $${\text{CH}}_{4}\;+\;4{\text{N}}_{2}\text{O }\to \;{\text{CO}}_{2}\;+\;4{\text{H}}_{2}\;+\;2{\text{H}}_{2}\text{O }\Delta {\text{G}}^{\text{ο}’}\;({\text{kJ mol}}^{-1})\;=\;-1230.14*$$

Despite no stoichiometric evidence was collected from these experiments, the parallel occurrence of AOM and N_2_O reduction prevailing in the ^13^CH_4_/N_2_O/PPHS treatment suggests that PPHS may play an important role on mediating these two microbial processes. No ^13^CO_2_ production was observed in experimental treatments lacking PPHS or N_2_O, further emphasizing the role of humic substances as electron shuttles for coupling AOM to N_2_O reduction (Figure 3).

### PPHS Redox Pattern During AOM Linked to N_2_O Reduction

**FIGURE 4:**
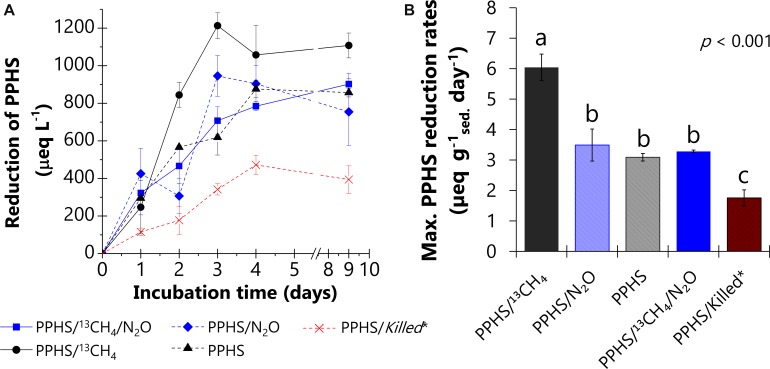
Reduction of Pahokee Peat Humic Substances (PPHS) under the different experimental conditions during the incubation time. PPHS were supplied in the oxidized form at the beginning of the incubation. Panel **(A)** shows the reduction of PPHS during incubation period. Panel **(B)** compares the maximum rates of PPHS reduction among the different experimental treatments based on the linear regressions of at least three sampling points during the period of the highest activity. Data represent the average from triplicate incubations ± standard error. *Killed controls contained the same concentrations of ^13^CH_4_ (∼4 mmol L^– 1^) and N_2_O (6.6 ± 0.4 mmol L^– 1^) as in the main experimental treatments. Statistically different treatments are represented with letters obtained via a one-way ANOVA and the Duncan *post hoc* test (95% percent confidence interval).

^∗^Further details on these thermodynamic calculations are included in Supporting Information (Supplementary Figure S3).

The redox state of PPHS was monitored during the observed AOM linked to N_2_O reduction in the 2nd cycle of incubation (Figure 4). As expected, microcosms containing ^13^CH_4_ and PPHS, but lacking N_2_O, displayed the maximum rates of PPHS reduction (6 ± 0.4 μeq g _sed._^–1^ day^–1^, Figure 4A) (mean ± SE, *n* = 3), while endogenous controls incubated in the absence of ^13^CH_4_ showed PPHS reduction rates ∼42% lower than the former experimental treatment. These findings confirm the utilization of ^13^CH_4_ as electron donor by humus-reducing microorganisms (humus-driven AOM, Eq. 3).

(3) $${\text{CH}}_{4}\;+\;4\text{Q }\cdot \;{\text{PPHS}}_{\text{ox}}\;+\;2{\text{H}}_{2}\text{O }\to \;{\text{CO}}_{2}\;+\;4{\text{QH}}_{2}\;\cdot \;{\text{PPHS}}_{\text{red }}\;\Delta {\text{G}}^{\text{ο}’}\;({\text{kJ mol}}^{-1})\;=\;-30\text{ to }-416.5*$$

**FIGURE 5:**
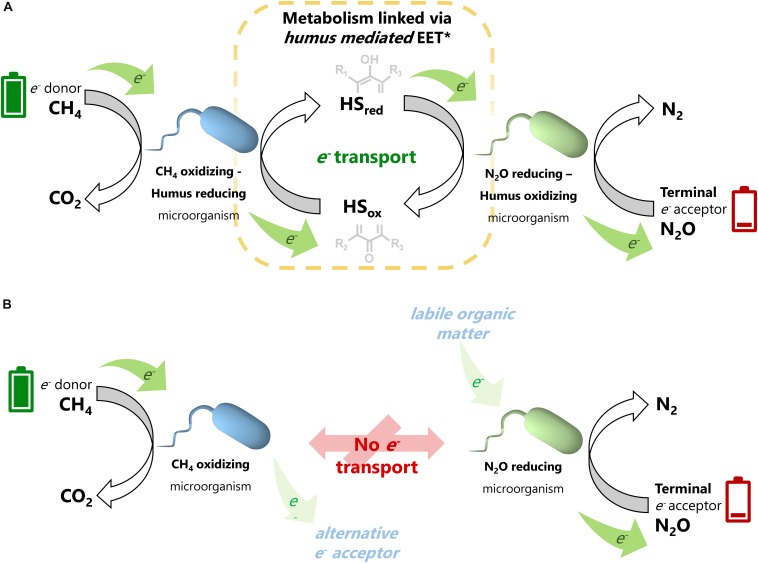
Schematic representation of anaerobic CH_4_ oxidation linked to N_2_O reduction mediated by the electron shuttling capacity of humic substances. Panel **(A)** illustrates the extracellular electron transfer process promoted by humic substances, which links the metabolic capabilities of anaerobic methane oxidizing and nitrous oxide reducing microbes. Full (green) batteries are a representation of the high content of reducing equivalents in CH_4_, which are taken by anaerobic methane oxidizing-humus reducing microorganisms and then taken from the reduced redox-active moieties (hydroquinones) by humus oxidizing-nitrous oxide-reducing microbes to reduce N_2_O, represented by an energy depleted (red) battery, into inert N_2_. In the absence of humic substances **(B)**, each process consuming CH_4_ and N_2_O could be independently fueled by an alternative electron donor or electron acceptor present at the wetland sediments (displayed in attenuated colors).

Moreover, microcosms amended with both ^13^CH_4_ and N_2_O in the presence of PPHS displayed the lowest PPHS reduction rates among all experimental treatments (3.28 ± 0.04 μeq g _sed._^–1^ day^–1^) (mean ± SE, *n* = 3) (Figure 4B). This confirms that PPHS_red_ produced via AOM were re-oxidized to PPHS_ox_ coupled to N_2_O reduction (Figure 5).

### Microbial Communities Potentially Involved in AOM Linked to N_2_O Reduction

#### Bacterial Taxa

**FIGURE 6:**
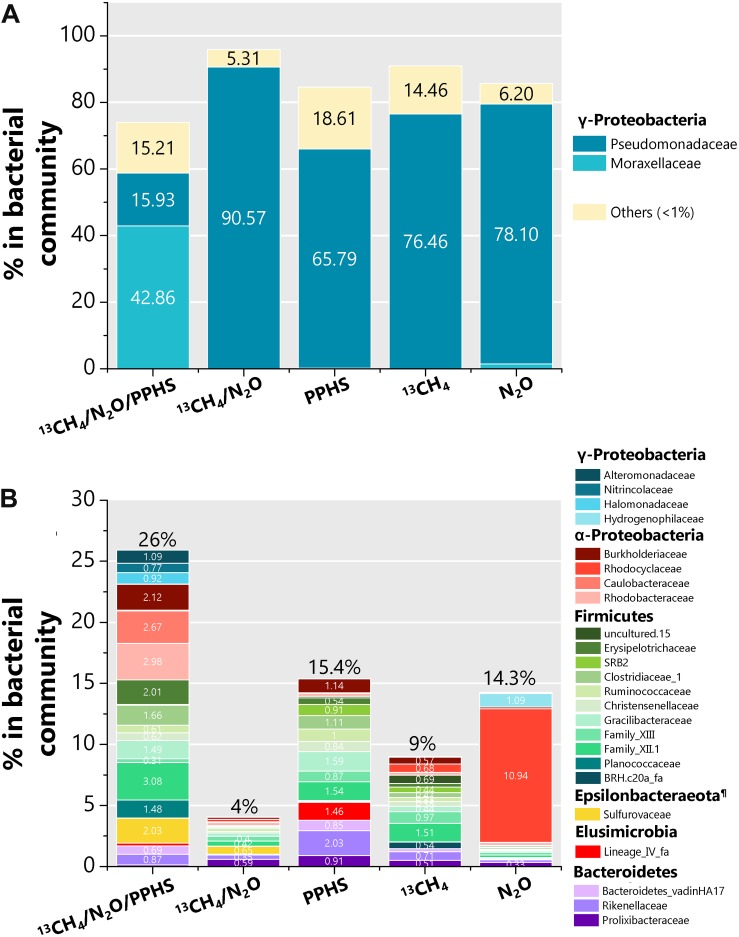
Composition of the bacterial communities found in selected experimental treatments at the end of the incubation period. Panel **(A)** displays those microbial taxa, which predominated the bacterial communities at the family level (>15% of abundance) as well as the summarized fraction of all families, which percentage in the whole bacterial community was lower than 1%. Panel **(B)** displays all bacterial families whose percentage in the bacterial community was between 1 and 15%. All data represent the average from two or three (^13^CH_4_/N_2_O treatment) genomic libraries sequenced. Each 16S rRNA library was generated from an independent DNA sample extracted from one biological replicate. ^¶^ The Epsilonbacteraeota were formerly known as the ε class of the Proteobacteria phylum.

According to 16S rRNA gene sequences performed at end of the incubation cycles, some bacterial groups considerably changed their relative abundance within the whole bacterial community under the selective conditions established in each experimental treatment (Figure 6). The most noticeable feature in the bacterial community in sediment incubations in which PPHS mediated AOM linked to N_2_O reduction was the increased relative abundance of a member of the *Moraxellaceae* family, identified as an *Acinetobacter* phylotype (accounting for 43% of the bacterial community, Figure 6A). The most predominant taxon observed in the remaining experimental treatments belongs to ɣ-Proteobacteria. However, the percentages of this member of *Pseudomonas* within each respective microbial community did not show any relationship with the presence of PPHS, CH_4_ or N_2_O during the experiments (Figure 6A).

Additional bacterial groups, which relative percentage was noticeably prominent in the ^13^CH_4_/N_2_O/PPHS experimental treatment respect to the controls (Figure 6B) belonged to the families *Alteromonadaceae* (ɣ-Proteobacteria), *Burkholderiaceae*, and *Caulobacteraceae* from the α-Proteobacteria, *Erysipelotrichaceae* and the family XII from the Firmicutes, as well as *Sulfurovaceae* from the Epsilonbacteraeota phylum (formerly ε-Proteobacteria).

#### Archaeal Taxa

**FIGURE 7:**
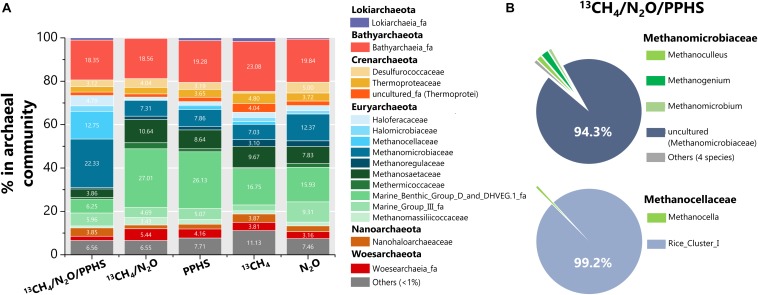
Composition of the archaeal communities found in selected experimental treatments at the end of the incubation period. Panel **(A)** shows the distribution of the whole archaeal sequences obtained through archaeal 16S rRNA ILLUMINA sequencing at family level amongst the experimental treatments. Panel **(B)** displays the analysis of Methanomicrobiaceae and Methanocellaceae in the ^13^CH_4_/N_2_O/PPHS treatment at the genus level. All data represent the average from the sequencing of two 16S rRNA libraries. Each library came from an independent DNA sample extracted from an experimental replicate.

Sequencing of archaeal 16S rRNA genomic libraries displayed high percentages of two families of the Euryarchaeota phylum in the ^13^CH_4_/N_2_O/PPHS treatment with respect to the experimental controls (Figure 7). *Methanocellaceae* and *Methanomicrobiaceae* families [99.2% and 94.3% dominated by the Rice Cluster I (RC-I) genera and an *uncultured* phylotype, respectively (Figure 7B)] were highly abundant as compared to the experimental control conditions. In the case of *Methanocellaceae* family, its abundance was remarkably higher (12.7%) than that found in the controls (0.06 to 1.8%), while *Methanomicrobiaceae* family approximately doubled its proportion with respect to the controls (22.3% vs. 7.3 to 12.4%, Figure 7A). Furthermore, important members of the archaeal community in all treatments include the Bathyarcheota phylum (∼20%), Marine Benthic Group D and DHVEG family (6–27%) as previously reported for this wetland sediment (Valenzuela et al., 2017). Previously reported AOM-performing archaea were detected at very low percentages in the archaeal communities of the wetland sediment. For instance, ANME-1 was only detected in the ^13^CH_4_/N_2_O/PPHS treatment in a 0.07% proportion of the archaeal community, while the abundance of ANME-3 ranged 0.05 to 0.11% in all experimental treatments. Moreover, Candidatus *Methanoperedens nitroreducens*, which has been related to AOM under denitrifying conditions (Haroon et al., 2013) was not detected.

## Discussion

The purpose of the present study was to evaluate the capacity of humic substances to mediate AOM coupled to the reduction of N_2_O by the microbiota present in a wetland sediment. The present study provides additional evidence of the important contribution of humic substances to suppress the emission of GHG in wetland sediments (Blodau and Deppe, 2012; Valenzuela et al., 2017, 2019). AOM linked to N_2_O reduction is a thermodynamically feasible reaction (see Eq. 2). Nevertheless, poor solubility of CH_4_ [∼19.7 mg CH_4_ L^–1^ @ 1 atm and 28°C (Payne, 2017)] in aquatic environments may be an important regulator of the process in natural ecosystems (Serra et al., 2006). The redox mediating capacity of humic substances may help to overcome these limitations by storing reducing equivalents derived from AOM and then channeling them toward the reduction of N_2_O. This is partly supported by the fact that both soluble and particulate NOM are effective redox mediators fueling several abiotic and microbial processes (Roden et al., 2010; Martinez et al., 2013).

Our results suggested that intrinsic NOM present in the studied wetland sediment promoted AOM linked to N_2_O reduction. First, there was significant N_2_O reduction coupled to the loss of the EDC of the NOM present in the wetland sediment when incubations were performed with N_2_O lacking PPHS and ^13^CH_4_ (Figure 1 and Table 1), pointing to the active role of reduced redox groups (e.g., hydroquinones) in the sediment’s NOM sustaining N_2_O reduction. Additionally, during the 1st cycle of simultaneous AOM and N_2_O reduction, the treatment lacking PPHS, but including both ^13^CH_4_ and N_2_O, showed higher N_2_O reduction than that observed in the treatment lacking PPHS and ^13^CH_4_ (Supplementary Figure S2). Although this evidence was not stoichiometric probably due to labile organic matter fueling N_2_O reduction, these initial findings suggest that intrinsic NOM might have played a role in AOM linked to N_2_O reduction. The role of humic substances in the proposed mechanism was further emphasized in incubations conducted with ^13^CH_4_/N_2_O/PPHS, which showed parallel AOM and N_2_O reduction (Figures 2, 3). Altogether, these results suggest the theoretical feasibility for this process to occur in nature, which according to our calculations is possible for the whole range of redox potentials (E^o^’) proposed for humic substances (−300 to +300 mV, Eq. 1) (Straub et al., 2000; Aeschbacher et al., 2010). Nevertheless, further studies are required to verify if the process prevails under natural conditions since the concentration of ^13^CH_4_ and N_2_O used in our incubations are much higher than those usually occurring in aquatic environments. Moreover, our incubations started several months after the sampling time, thus the inoculum might have not reflected the actual microbial community of the studied wetland sediment. Despite these limitations, our study suggests that humic substances could mediate AOM linked to N_2_O reduction in wetland sediments, which constitutes a new pathway interconnecting the C and N cycles in these ecosystems. Humic substances have previously been reported to link the C and N biogeochemical cycles by anaerobic ammonium oxidation coupled to microbial reduction of NOM in marine sediments (Rios-Del Toro et al., 2018b).

The current knowledge on anaerobic methanotrophic microorganisms that are able to link the C and the N cycles through AOM driven by oxidized N compounds, describes the mandatory utilization of NO_3_^–^ (by Candidatus *Methanoperedens nitroreducens*, an ANME-2d affiliated archaeon) or NO_2_^–^ (by Candidatus *Methylomirabilis oxyfera*, a bacterium) as TEA (Ettwig et al., 2010; Zhu et al., 2012; Haroon et al., 2013). Nevertheless, there are no kinetic or genetic studies showing that these microbes could use N_2_O as TEA, either by themselves or by a syntrophic relationship with another microbial partner to oxidize CH_4_.

A previous study demonstrating the simultaneous consumption of CH_4_ and N_2_O in an artificial wetland proved that this coupled process stimulated the activity of aerobic methanotrophs (Cheng et al., 2019). However, no parallel stimulation of *nosZ* enzymatic activity could be identified; consequently, the mechanisms for the coupled reaction remained unknown. In the present work, we provide several clues indicating that humic substances can mediate the coupling between the methanotrophs and N_2_O reducers, which would expand our understanding of the processes driven by the microbial communities in wetland sediments (Figure 5).

### Microbial Communities Potentially Involved

The bacterial taxa whose relative abundance increased in the treatment displaying simultaneous ^13^CH_4_ and N_2_O consumption mediated by PPHS, agreed with previously reported denitrifying and non-denitrifying N_2_O reducing bacteria, which have been described in distinct ecosystems at the genus taxonomical level (Figure 6B) (Conthe et al., 2018; Hallin et al., 2018). Likewise, one bacterial taxum, which stood out due to its important relative abundance within the 16S libraries under the ^13^CH_4_/N_2_O/PPHS conditions was a ɣ-proteobacterial phylotype from the *Acinetobacter* genus (Figure 6A). Previous studies, have shown the involvement of *Acinetobacter* species in the N cycle due to their capacity to accomplish heterotrophic nitrification-aerobic denitrification (Chen et al., 2019; Wen et al., 2019), whilst other species have been reported as heterotrophic and autotrophic denitrifiers (Lee et al., 2013; Pishgar et al., 2019). Two works reported by Su et al. (2015, 2016), showed that *Acinetobacter* sp. strain SZ28 was able to accomplish NO_3_^–^ and N_2_O reduction coupled to Mn^2+^, Fe^2+^, and S^2–^ oxidation. These authors also documented the capacity of strain SZ28 to employ several organic compounds, including humic substances, as an energy source. Thus, based on the body of evidence demonstrating the respiratory and metabolic versatility of *Acinetobacter* species, we hypothesize that some species of this microbial taxon could have been potentially involved in N_2_O reduction using reduced redox groups in PPHS_red_ as electron donor. Nevertheless, we cannot dismiss the potential involvement of other N_2_O reducers in the process since the diversity of microbes possessing this feature is as wide as the diversity of humus-oxidizing microorganisms (Lovley et al., 1999; Martinez et al., 2013); however, future research must be done in order isolate and characterize the microorganisms conducting this process.

From the archaeal counterpart, only two taxa showed considerable increase within the microbial community in the complete treatment including ^13^CH_4_/N_2_O/PPHS: an uncultured genus of the *Methanomicrobiaceae* family, and the RC-I genus from the *Methanocellaceae* (Figures 7A,B). By using H_2_, formate and CO_2_ as substrates for methanogenesis (Hunger et al., 2011), the RC-I cluster has been proposed as the most important archaea controlling CH_4_ emissions from paddy soils (Großkopf et al., 1998; Erkel et al., 2006). Despite this, no methanogenic activity was detected in our incubations under any of the experimental conditions tested. Archaeal members of the RC-I cluster have previously been detected in wetland and marsh ecosystems (Lin et al., 2017; Xiao et al., 2017), as well as in rice paddies. These organotrophic systems contain high amounts of endlessly decomposing NOM, which prevail under anoxic conditions due to flooding, thus creating the proper niche for microbes to perform the redox reactions involved in AOM coupled to N_2_O reduction, mediated by humic substances (Figure 5). Although to the best of our knowledge, the RC-I archaeal group has not been reported to perform either AOM or humus-reducing activities, Bao et al. (2016) showed that NO_3_^–^ addition promoted a positive response in RC-I archaea in terms of the expression the *mcrA* gene (a molecular marker of methanogenesis and/or methanotrophy). This effect was also linked to diminished CH_4_ production and given the evidence provided in the present study, a possible connection involving the RC-I type of archaea between denitrification and methanotrophy must be addressed in future studies.

Regarding the uncultured *Methanomicrobiaceae* phylotype, which comprised ∼20% of the total archaeal community, in addition to the numerous reports on its role as CH_4_ producer (Kröber et al., 2009), a recent study showed that the *Methanobacterium* genera, which also belongs to the *Methanomicrobiaceae* family, drove AOM coupled to ferrihydrite reduction with humic substances as electron shuttle (He et al., 2019). These authors demonstrated how this microorganism oxidized CH_4_ to propionate and proposed that this intermediate was then taken by a bacterial partner (potentially a *Desulfovibrio* species) to produce siderite (a ferrous iron carbonate). Previous reports have described the syntrophic activity of anaerobic methanotrophic archaea in partnership with bacteria to reduce TEAs, such as SO_4_^2–^ (Boetius et al., 2000). Here, we propose a novel syntrophic process mediated by humic substances in which the RC-I cluster and/or an uncultured member of the *Methanomicrobiaceae* family could have coupled metabolic capabilities with a bacterial member, such as *Acinetobacter*, to perform AOM linked to N_2_O reduction via an EET mechanism mediated by humic substances (Figure 5).

## Conclusion

The present study showed several lines of evidence indicating that humic substances mediate an EET process in which AOM is linked to N_2_O reduction in microcosms derived from coastal wetland sediments. These results further emphasize the relevant role that humic substances could play to prevent the emission of GHG from organotrophic environments and provide insights into the potential of their redox active groups as a metabolic linking agent for connecting the C and N cycles. However, further studies are needed to verify if this process prevails under natural conditions.

## Data Availability Statement

The accession numbers of the sequences in this work (SAMN13002101 to SAMN13002126) were deposited in the GenBank Sequence Read Archive under BioProject number PRJNA576687.

## Author Contributions

EV and FC conceived the research, designed the experimental set-up, and wrote the manuscript with input from all the authors. CP-L and EV performed the experiments. EV, SC-F, and NL-L coordinated the molecular biology research, which was technically performed by EV, NG-H, and CP-L. NL-L performed the bioinformatic analyses and interpreted the results along with EV.

## Conflict of Interest

The authors declare that the research was conducted in the absence of any commercial or financial relationships that could be construed as a potential conflict of interest.
