# Supplementary material for: Humic Substances Mediate Anaerobic Methane Oxidation Linked to Nitrous Oxide Reduction in Wetland Sediments
Source: Front Microbiol. 2020 Apr 15;11:587. doi: 10.3389/fmicb.2020.00587 (PMC7174564; doi:10.3389/fmicb.2020.00587)
Supplement: Supplementary file 1 [file Data_Sheet_1.docx]

**-Supporting Information-**

**HUMIC SUBSTANCES MEDIATE ANAEROBIC METHANE OXIDATION LINKED TO NITROUS OXIDE REDUCTION IN WETLAND SEDIMENTS**

**Running Title:** AOM linked to N_2_O reduction

Edgardo I. Valenzuela^1^, Claudia Padilla-Loma^1^, Nicolás Gómez-Hernández^2^, Nguyen E. López-Lozano^1^, Sergio Casas-Flores^2^, Francisco J. Cervantes^3*^

1. IPICYT, División de Ciencias Ambientales, San Luis Potosí, Mexico.
2. IPICYT, División de Biología Molecular, San Luis Potosí, Mexico.
3. Laboratory for Research on Advanced Processes for Water Treatment, Engineering Institute, Campus Juriquilla, Universidad Nacional Autónoma de México (UNAM), Querétaro, Mexico

***Correspondence:**

Corresponding Author

[fcervantesc@iingen.unam.mx](mailto:fcervantesc@iingen.unam.mx)

**Table S1.** Experimental conditions implemented in incubations to demonstrate N_2_O reduction with reduced *Pahokee Peat* Humic Substances (PPHS_red_) as electron donor.

| **Treatment name** | **Symbol** | **Reduced PPHS, 1000 mg L^-1^)** | **Oxidized PPHS, 1000 mg L^-1^)** | **N_2_O (4 mL)** | **Autoclave (3 cycles) + chloroform (10%, v/v)** |
| --- | --- | --- | --- | --- | --- |
| PPHS_red_/N_2_O | **♦** | **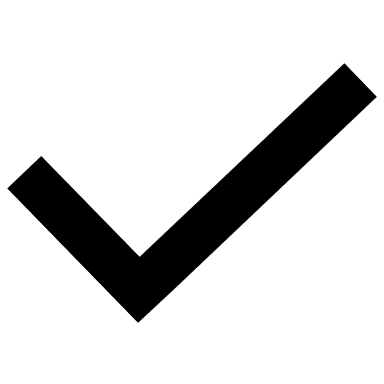** |  | **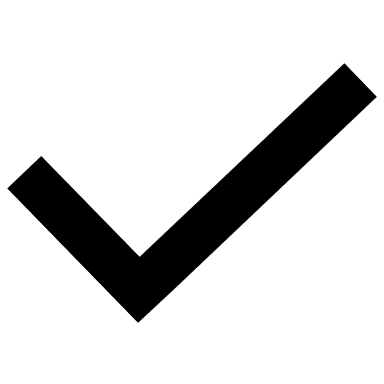** |  |
| PPHS_ox_/N_2_O | **■** |  | **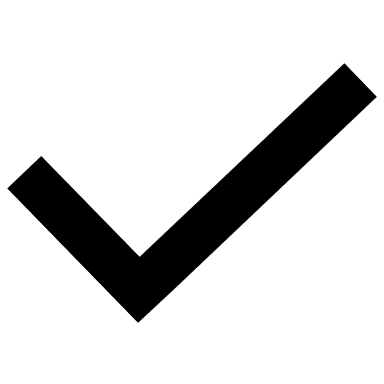** | **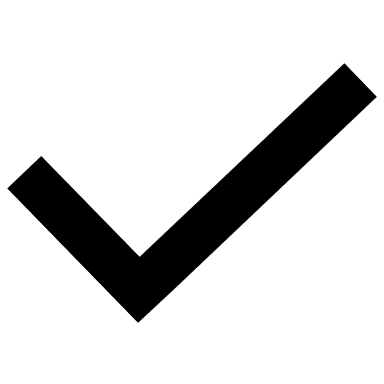** |  |
| PPHS_red_ (*endogenous*)^¶^ | **▲** | **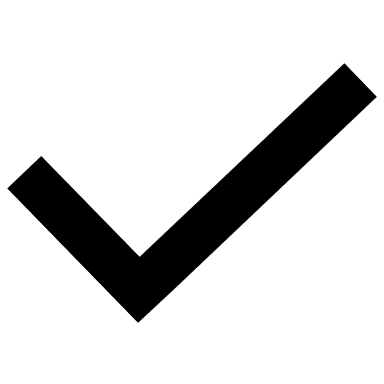** |  |  |  |
| PPHS_red_/*Killed** | **x** | **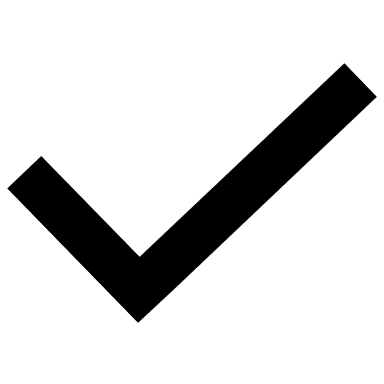** |  | **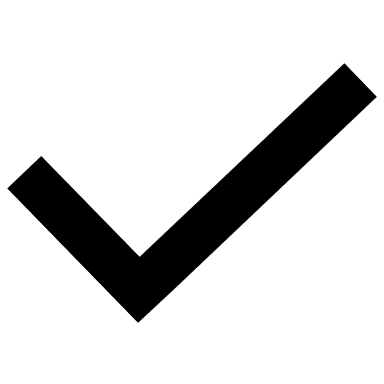** | **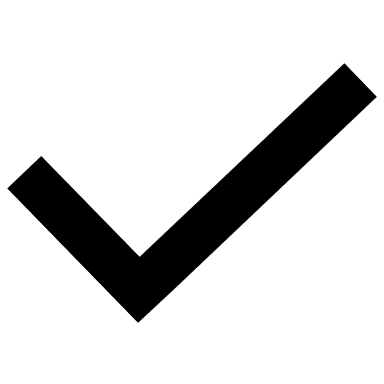** |
| N_2_O | **●** |  |  | **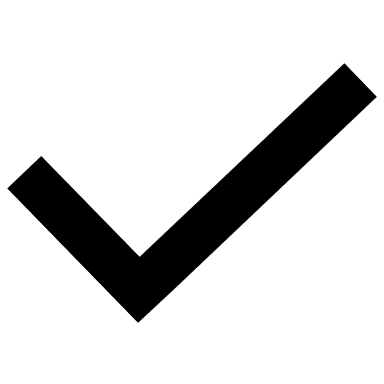** |  |

^¶^*endogenous* refers to those controls without the addition of N_2_O acting as electron acceptor, therefore these microcosms’ atmosphere was only composed of argon (99.9% purity, Praxair). **Killed* controls headspace was spiked with the same volumes of N_2_O added in the microbially active treatments.

**Table S2.** Description of the experimental conditions and controls implemented in incubations demonstrating CH_4_ and N_2_O simultaneous consumption mediated by *Pahokee Peat* Humic Substances (PPHS).

| **Treatment name** | **Symbol** | ***Pahokee Peat* Humic Substances, 500 mg L^-1^)** | **N_2_O (4 mL)** | **^13^CH_4_ (2 mL)** | **Autoclave (3 cycles) + chloroform (10%, v/v)** |
| --- | --- | --- | --- | --- | --- |
| PPHS (*endogenous*)^¶^ | **▲** | **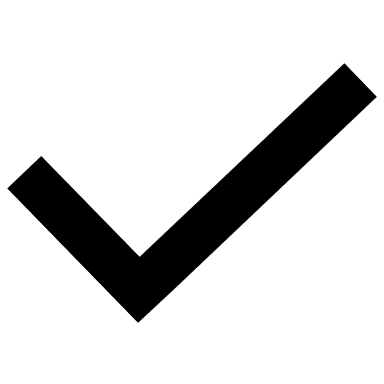** |  |  |  |
| PPHS/^13^CH_4_/N_2_O | **■** | **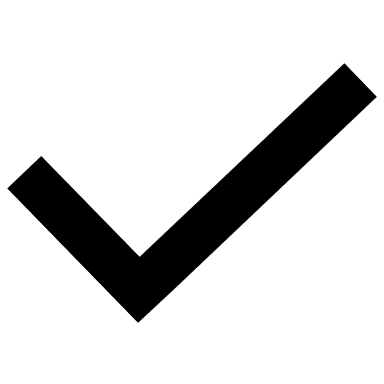** | **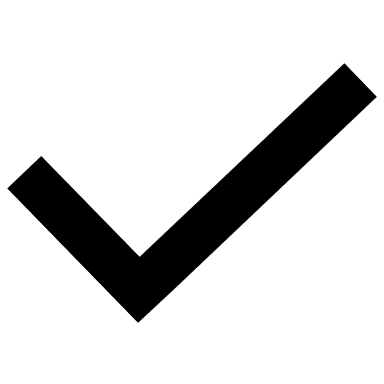** | **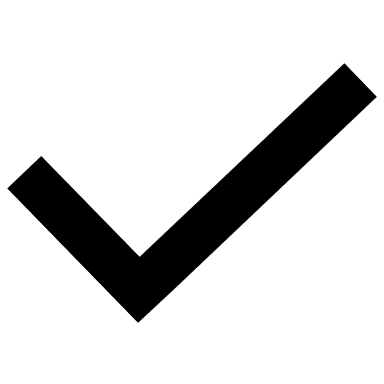** |  |
| PPHS/N_2_O | **♦** | **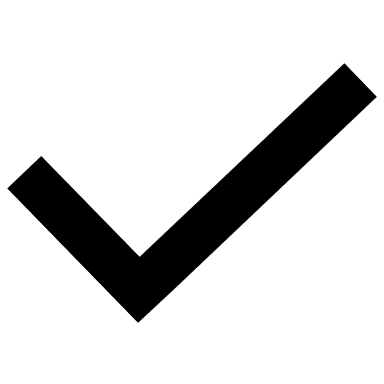** | **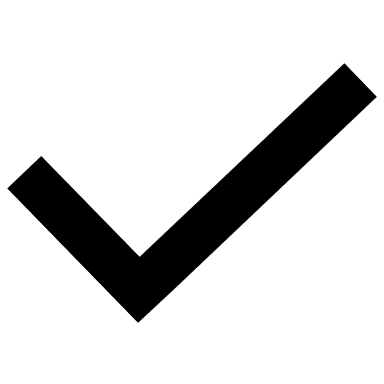** |  |  |
| PPHS/^13^CH_4_ | **●** | **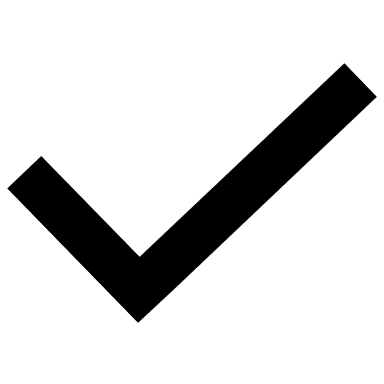** |  | **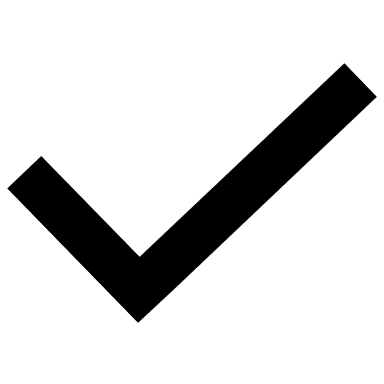** |  |
| PPHS/*Killed** | **x** | **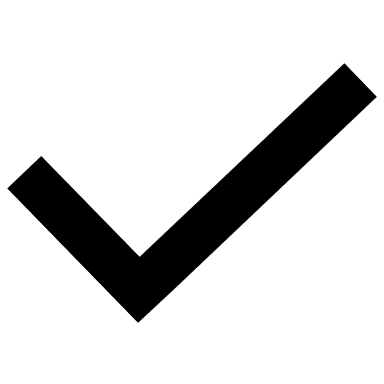** | **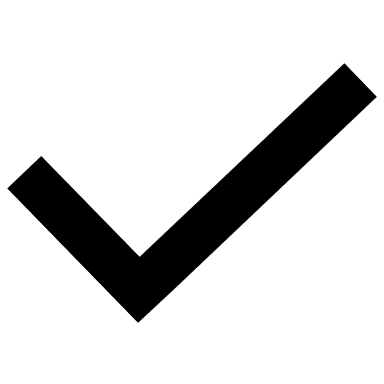** | **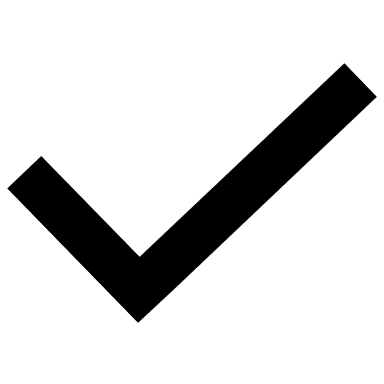** | **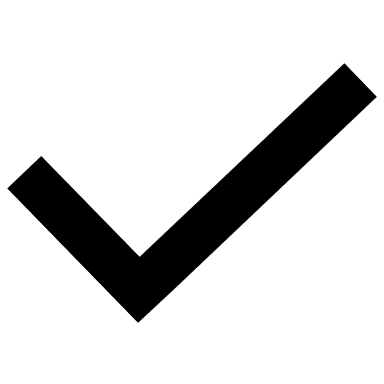** |
| Sediment (*endogenous*)^¶^ | **▲** |  |  |  |  |
| ^13^CH_4_/N_2_O | **■** |  | **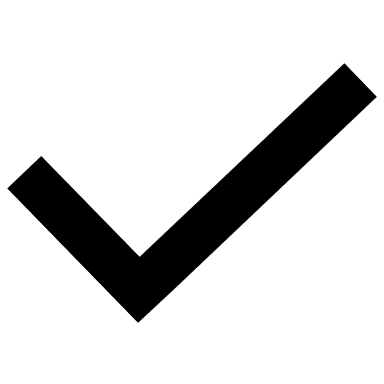** | **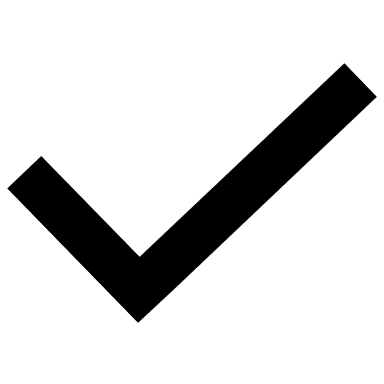** |  |
| N_2_O | **♦** |  | **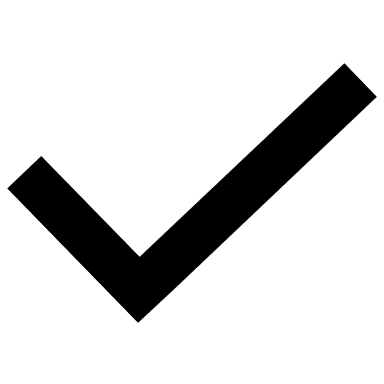** |  |  |
| ^13^CH_4_ | **●** |  |  | **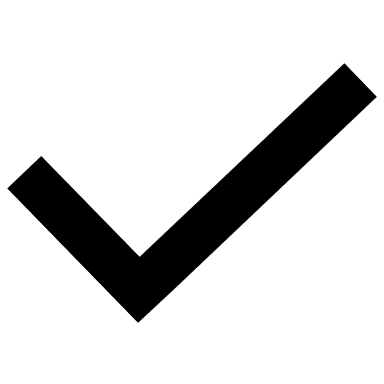** |  |
| *Killed** | **x** |  | **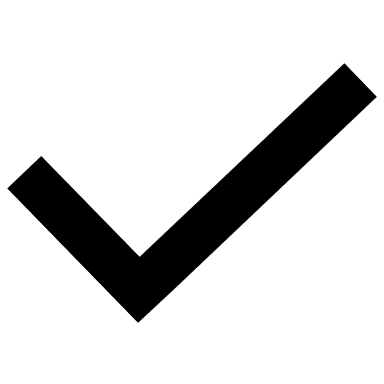** | **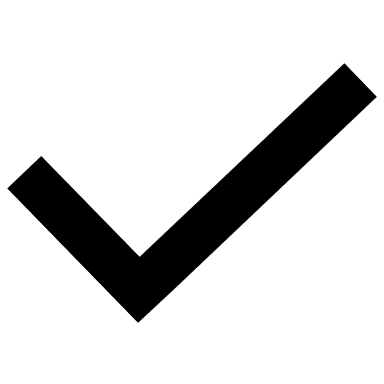** | **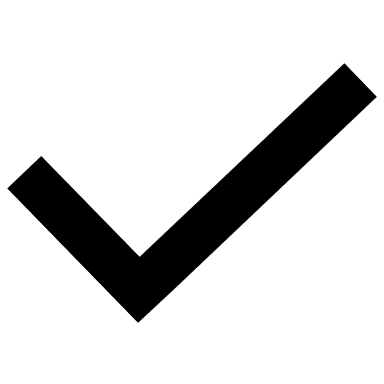** |

^¶^*endogenous* refers to microcosms without the addition of gases acting as electron donor or acceptor (^13^CH_4_ or N_2_O), therefore these microcosms’ atmosphere was only composed of argon (99.9% purity, Praxair). **Killed* controls headspace was spiked with the same volumes of ^13^CH_4_ and N_2_O added in the microbially active treatments.

**Figure S1. GC-MS chromatograms showing qualitative evidence of the reduction of ^15^N_2_O to ^30^N_2_ (^15,15^N_2_) with PPHS_red_ as electron donor.** Superior panels display signals extracted for the ion mass equivalent to 30 in which the signal for ^30^N_2_ can be found at the retention time of ~1.8 min. Inferior panels display the chromatograms extracted for the ion mass equivalent to 46 in which the signal for ^30^N_2_O can be found at the retention time of ~2.3 min.


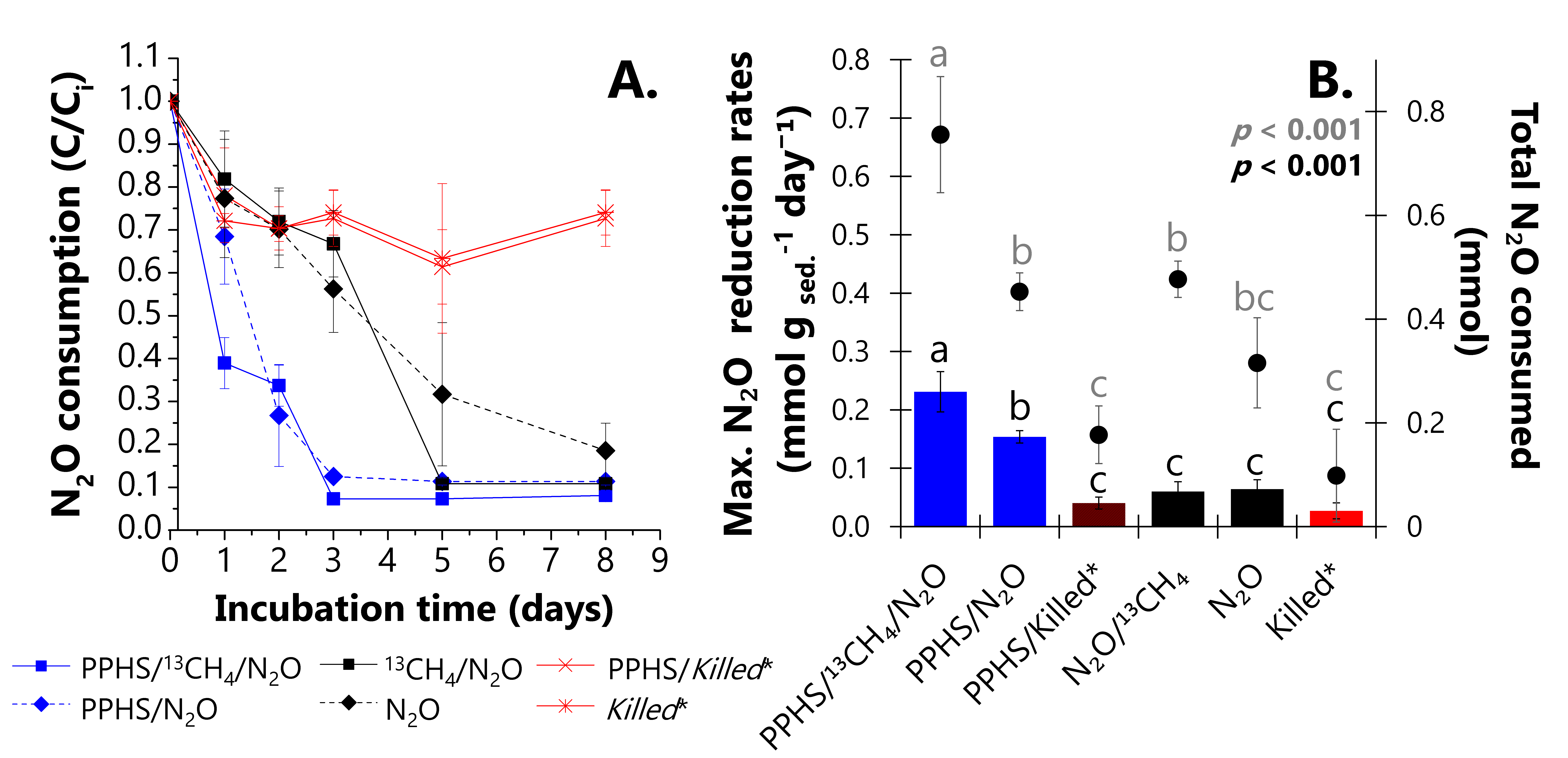


**Figure S2. Nitrous oxide reduction promoted by *Pahokee Peat* Humic Substances (PPHS) acting as electron shuttle and ^13^CH_4_ as electron donor during the first cycle of incubation. Panel A** depicts the normalized (concentration / initial concentration, C/C_i_) kinetics of N_2_O consumption with and without PPHS as electron shuttle. **Panel B** shows the maximum N_2_O reduction rates (bars, left axis) based on the linear regressions of at least three sampling points during the period of highest activity. The net amount of N_2_O depleted after 8 days of incubation is shown in the right axis (**•** symbols). Data represent the average from triplicate incubations ± standard error. *Killed controls contain the same concentration of ^13^CH_4_ and N_2_O as in the main experimental treatments. Statistically different treatments are represented with letters obtained via a one-way ANOVA and the Duncan post hoc test (95% percent confidence interval).

**Figure S3. Standard Gibbs free energy (ΔG°`, considering pH = 7) of the humic substances (HS)-dependent or -mediated reactions examined in this work.** Calculations were made according to Nernst equation considering the reduction of N_2_O to N2 (**Eq. 1 and 3**), the oxidation of ^13^CH_4_ to ^13^CO_2_ (**Eq. 2 and 3**), and the whole range of standard redox potentials (E°`) reported for HS (data taken from references)^1,2^:

**ΔG°` = - n * F * ΔE°`**

**Where:**

**n** = number of electrons transferred during the redox reaction per mol of N_2_O or ^13^CH_4_ = 2 or 8, respectively.

**F** = Faraday constant = 96. 56 kJ/V-mol

ΔE°` = Difference in standard redox potential between electron acceptor and electron donor

**For Equation 1:**

ΔE°`= (E°`N_2_O/N_2_) – (E°`HS_ox_/HS_red_)

E°` for Humic Substances = ^¶^from -0.3 V to +0.3 V

E°` for couple N_2_O/N2 = +1355 V

**For Equation 2:**

ΔE°`= (E°`HS_ox_/HS_red_) – (E°`CH_4_/CO_2_)

E°` for Humic Substances = ^¶^from -0.3 V to +0.3 V

E°` for couple CH_4_/CO_2_ = -240 V

**For Equation 3:**

ΔE°`= (E°`N_2_O/N_2_) – (E°`CH4/CO2)

E°` for couple N_2_O/N_2_ = +1355 V

E°` for couple CH_4_/CO2 = -240 V

**References**:

(1) Straub, K. L.; Benz, M.; Schink, B. Iron metabolism in anoxic environments at near neutral pH. *FEMS Microbiol. Ecol.* **2000**, *34* (3), 181–186.

(2) Aeschbacher, M.; Vergari, D.; Schwarzenbach, R. P.; Sander, M. Electrochemical analysis of proton and electron transfer equilibria of the reducible moieties in humic acids. *Environ. Sci. Technol.* **2011**, *45* (19), 8385–8394.
